# Mechanism and role of MCP-1 upregulation upon chikungunya virus infection in human peripheral blood mononuclear cells

**DOI:** 10.1038/srep32288

**Published:** 2016-08-25

**Authors:** Mariana Ruiz Silva, Heidi van der Ende-Metselaar, H. Lie Mulder, Jolanda M. Smit, Izabela A. Rodenhuis-Zybert

**Affiliations:** 1 Department of Medical Microbiology, University of Groningen and University Medical Center Groningen, 9700 RB, Groningen, The Netherlands

## Abstract

Monocyte chemoattractant protein-1 (MCP-1/CCL2)-mediated migration of monocytes is essential for immunological surveillance of tissues. During chikungunya virus (CHIKV) infection however, excessive production of MCP-1 has been linked to disease pathogenesis. High MCP-1 serum levels are detected during the viremic phase of CHIKV infection and correlate with the virus titre. *In vitro* CHIKV infection was also shown to stimulate MCP-1 production in whole blood; yet the role and the mechanism of MCP-1 production upon infection of human peripheral blood mononuclear cells remain unknown. Here we found that active CHIKV infection stimulated production of MCP-1 in monocytes. Importantly however, we found that communication with other leukocytes is crucial to yield MCP-1 by monocytes upon CHIKV infection. Indeed, blocking interferon-α/β receptor or the JAK1/JAK2 signalling downstream of the receptor abolished CHIKV-mediated MCP-1 production. Additionally, we show that despite the apparent correlation between IFN type I, CHIKV replication and MCP-1, modulating the levels of the chemokine did not influence CHIKV infection. In summary, our data disclose the complexity of MCP-1 regulation upon CHIKV infection and point to a crucial role of IFNβ in the chemokine secretion. We propose that balance between these soluble factors is imperative for an appropriate host response to CHIKV infection.

Chikungunya virus (CHIKV) is the causative agent of Chikungunya fever (CHIKF), an acute and an excruciatingly painful, musculoskeletal illness. CHIKV is a positive-sense, single-stranded RNA virus belonging to *Alphavirus* genus within the *Togaviridae* family. During the last decade, outbreaks of CHIKV have occurred among islands in the Indian Ocean, Australia, Southeast Asia, Africa, and India. Since December 2013 more than 1.5 million suspected and 75 thousand confirmed cases have been reported in the Americas^1^. CHIKF symptoms develop 3–5 days after infection and usually include fever, joint and muscle pain, rash, nausea and headache. In up to 57% of the cases, musculoskeletal pain may persist for years^2^,^3^,^4^. Rarely, fatal cases occur and this is typically in patients with underlying conditions^5^,^6^,^7^,^8^,^9^. The host’s innate immune response plays an important role in the control as well as the pathogenesis of CHIKV infection. Type-I interferon (IFN) signalling is an important factor that determines susceptibility to CHIKV severe disease^10^. High levels of type-I IFN, interleukin 1 beta (IL-1β), interleukin 6 (IL-6), monocyte chemoattractant protein (MCP-1/CCL2) and tumor necrosis alpha (TNF-α) in plasma of CHIKV patients correlate with high viral titters and severe disease^11^,^12^,^13^,^14^,^15^. Although the exact mechanism underlying musculoskeletal illness is not entirely understood, *in vivo* studies indicate a pathogenic role of soluble immune mediators and tissue-infiltrating monocytes^11^,^15^,^16^,^17^,^18^,^19^. For example, Labadie *et al.* found extensive macrophage infiltration in tissues of CHIKV-infected macaques and Gardner *et al.* showed that arthritic symptoms were associated with foci of inflammatory cells infiltrates, mainly monocytes, macrophages and natural killers, in synovial tissue of C57BL/6 mice infected with CHIKV^16^,^17^. Indeed, excessive production of factors involved in migration and activation of immune cells, such as IL-6, interferon gamma (IFN-γ), and MCP-1, in infected tissues could explain the features observed in CHIKV pathogenesis.

MCP-1 is encoded by the ccl2 gene and is produced in several cell types, including macrophages and fibroblasts^20^. Stimulation can occur directly through activation of pattern recognition receptors (PRR) and/or by cytokines such as, *inter alia,* IFNβ, IL-6 and TNFα^21^,^22^,^23^,^24^,^25^. MCP-1 acts as a chemo-attractant for monocytes as well as some other immune cells such as memory T lymphocytes and natural killer cells^20^. MCP-1-mediated migration of monocytes from the blood stream across the vascular endothelium is essential for routine immunological surveillance of tissues, as well as in response to inflammation^26^. Indeed, MCP-1 knockout mice have impaired monocyte recruitment following intraperitoneal thioglycolate administration, as well as in response to viral infection including influenza A virus, coronavirus and West Nile virus (WNV)^27^,^28^,^29^,^30^. Similarly, MCP-1 receptor knockout mice show increased cellular infiltration and develop more severe disease than wild type mice following infection with influenza virus, WNV and CHIKV^31^,^32^,^33^. On the other hand, inhibition of MCP-1 synthesis with use of bindarit protects against CHIKV-induced bone loss, arthritis and myositis in a mouse model of acute CHIKV disease^34^,^35^.

Despite the dual role of MCP-1 in CHIKV-mediated disease, our understanding of how the chemokine is controlled in the course of infection is limited. High MCP-1 serum levels are detected during in the acute phase of the infection in humans and laboratory animals, and coincide with the viremic period^4^,^17^,^36^. *In vitro,* CHIKV infection in whole blood stimulates MCP-1 production^37^, although the source and the mechanism of MCP-1 upregulation upon the infection remain elusive. Here, we deciphered the source and mechanism of MCP-1 upregulation during CHIKV infection in human peripheral blood mononuclear cells (PBMCs).

## Results

### Rapid MCP-1 production during CHIKV infection in PBMCs

**Figure 1:**
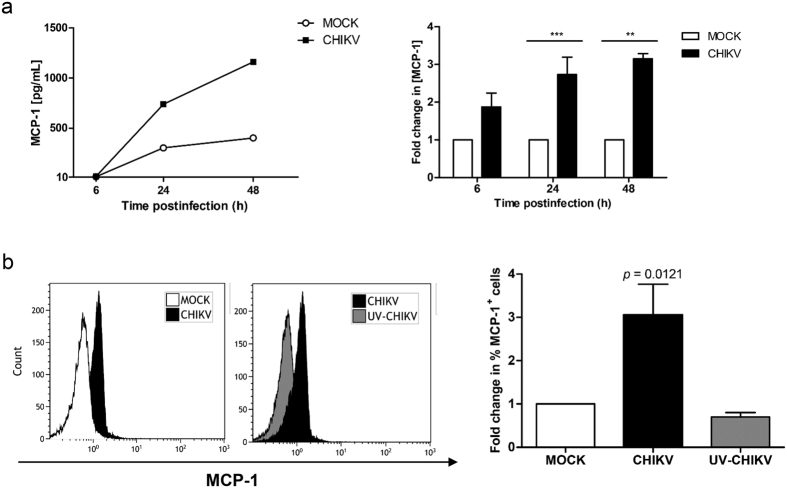
MCP-1 production during CHIKV infection in PBMCs. (**a**) Left, time course analysis of MCP-1 levels in supernatant of mock- and CHIKV-infected PBMCs, representative results from one donor. Right, fold change in MCP-1 concentration in the supernatant of CHIKV-infected PBMCs. At least three different donors per time point were analysed, n ≥ 3. Error bars show s.e.m. Two-way ANOVA with Bonferroni’s multiple comparison test was used for statistical analysis. (**b**) Upregulation of MCP-1 expression at 24 hpi was observed in response to replication competent CHIKV but not to UV-inactivated CHIKV (UV-CHIKV). Bars represent mean fold change over mock-infected cells + s.e.m. One-way ANOVA with Bonferroni’s multiple comparison test was used for statistical analysis. At least three different donors were analysed, n ≥ 3.

First, we confirmed that PBMCs secrete MCP-1 upon CHIKV infection. To this end, cells were infected with CHIKV-LR Opy-1 strain at a multiplicity of infection (MOI) of 1, 5 or 10. MCP-1 levels were measured over time in the cell supernatants (SN) while the cells were harvested for intracellular MCP-expression analysis at 24 hpi. Figure 1A shows a representative time course of MCP-1 levels following CHIKV infection (left panel) and an average of the maximum increase of MCP-1 scored for all donors at different time points (right panel) in the cell supernatant. CHIKV stimulated a rapid increase in MCP- production, with initial chemokine detectable levels at 6hpi. Despite some variability among donors in the MCP-1 basal expression, CHIKV infection increased the levels of MCP-1 on average 2- to 3-fold between 24–48 hpi. Intracellular staining of MCP-1 confirmed the increase in expression of MCP-1 following CHIKV infection (Fig. 1b). Also, and in line with previous studies^37^, non-replicative, UV-inactivated CHIKV (UV-CHIKV) did not trigger an increase in MCP-1 expression inferring that CHIKV replication is required for MCP-1 induction in PBMCs.

### Monocytes are the primary source of MCP- 1 during CHIKV infection in PBMCs

We next sought to disclose the cellular source of MCP-1 following CHIKV infection of PBMCs. Within blood, CD14^+^ monocytes are believed to be the major source of MCP-1^38^. Nonetheless, Her *et al.* reported that monocytes do not produce MCP-1 following CHIKV infection^37^. To elucidate the source of the chemokine upon CHIKV infection, mock and CHIKV-infected PBMCs were subjected to flow cytometric analysis as described in Methods section.

**Figure 2:**
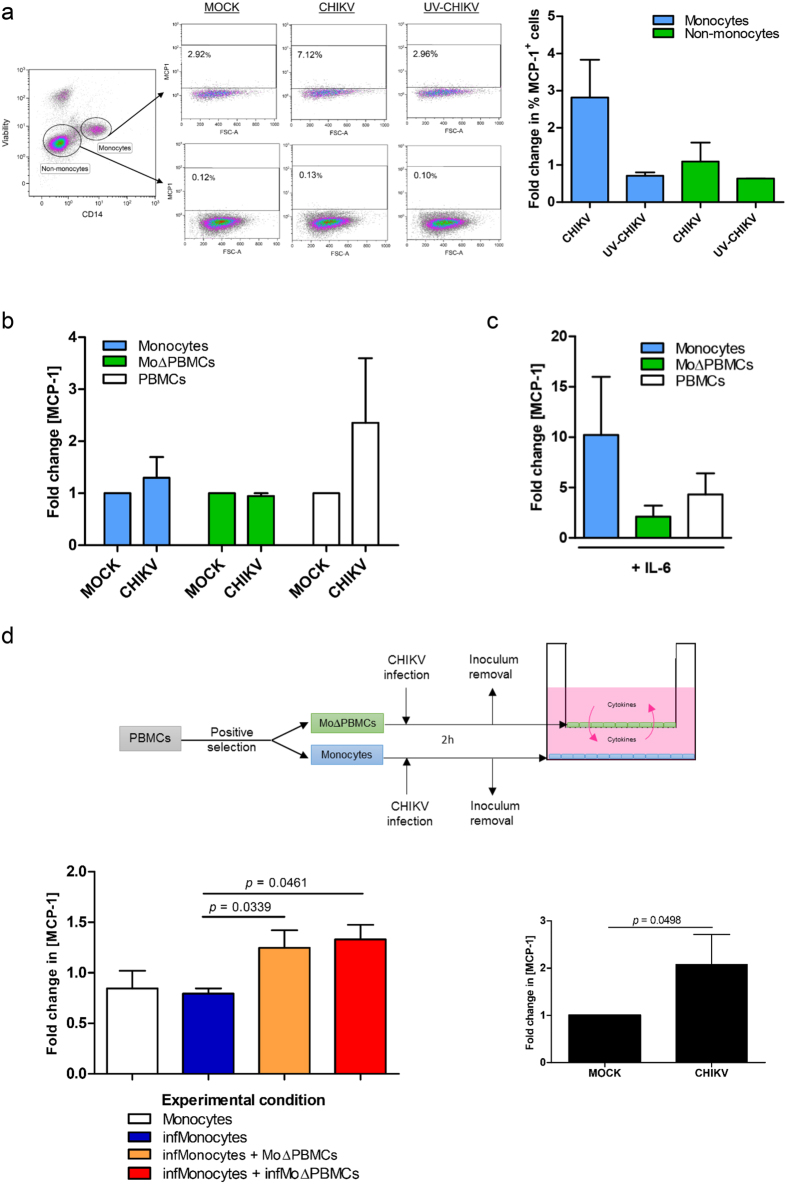
Monocytes are the primary source of MCP-1 among PBMCs in response to CHIKV infection however they require other cells to be able to produce it. (**a**) CHIKV-induced MCP-1 expression was found primarily in monocytes (defined as CD14^+^ cells). Bars represent mean + s.e.m., n = 3. (**b**) Infection of monocyte-depleted PBMCs (Mo∆PBMCs) or monocytes did not result in MCP-1 production 24 hpi. Infection of PBMCs from the same donors stimulated MCP-1 secretion. Bars represent mean + s.e.m., n = 3. (**c**) Monocytes, Mo∆PBMCs and PBMCs produced MCP-1 in response to IL-6 stimulation. Bars show mean fold increase over non-stimulated cells + s.e.m., n ≥ 3. (**d**) Transwell co-culture of Mo∆PBMCs with infected monocytes (infMonocytes) restored the capacity of the latter to secrete MCP-1. The right panel shows MCP-1 production of PBMCs from the same donors used in the transwell co-culture experiments. Bars represent mean fold increase over mock-infected (Monocytes + Mo∆PBMCs) + s.e.m., n = 3.

As CHIKV is known to cause cytopathic effects in some cells, we first assessed the viability of the cells following infection and its effect on CD14 expression at 24 hpi. As shown in Supplementary Fig. S1, we did not observe changes in cell viability or CD14 expression as a result of CHIKV infection. At 48 hours post infection levels of CD14 decreased in all experimental conditions (data not shown). Next, we gated the CD14^+^ (monocytes) vs CD14^−^ (non-monocytes) cells and compared MCP-1 expression levels following CHIKV infection. Surprisingly, increased MCP-1 expression levels were found following active CHIKV infection in monocytes (Fig. 2a), suggesting that these cells are the source of MCP-1 production. Monocytes have been also proposed to be the cellular vehicle for virus dissemination^4^,^16^,^37^ and therefore we aimed to investigate whether intracellular MCP-1 expression was restricted to only infected monocytes. Accordingly, we performed flow cytometric analysis to search for CHIKV E2 and MCP-1-positive PBMCs. Disappointingly; the frequencies of CHIKV^+^ monocytes were negligibly low at conditions with high MCP-1 expression. At MOIs high enough to detect convincing numbers of CHIKV^+^ monocytes (MOI 50 and higher, data not shown) however, we did not observe increase in MCP-1 secretion (nor expression), signifying that infection at such high MOI changes cytokine pattern considerably^39^.

To verify that CD14^+^ cells are indeed the main source of MCP-1 during CHIKV infection in PBMCs, we compared it to the levels of MCP-1 secreted by monocytes and monocyte-depleted PBMCs (MoΔPBMCs) from the corresponding donors (Fig. 2b). Counterintuitively however, no MCP-1 stimulation was observed upon infection of monocytes (Fig. 2b) at any MOI tested (Supplementary Fig. S2). Nonetheless, depletion of monocytes (on average 96% CD14^+^ cells was depleted) abrogated CHIKV-mediated secretion of the chemokine (Fig. 2b). Incubation of non-infected monocytes with the SN of infected MoΔPBMCs also did not stimulate MCP-1 production (not depicted), confirming that infection of monocytes was imperative to lead to the production of this chemokine. Addition of IL-6, used as a positive control, stimulated MCP-1 production in all cell preparations (Fig. 2c), indicating that positive selection did not affect the capacity of the cells to produce MCP-1 and was not the reason for the lack of MCP-1 secretion by infected monocytes. Thus far, these results implied that presence of other leukocytes was crucial for the chemokine production in the context of CHIKV infection. To test this, infected monocytes were cultured in the presence or absence of non-infected or infected MoΔPBMCs in a transwell co-culture system. In agreement with the data presented in Fig. 2b, neither monocytes nor MoΔPBMCs secreted MCP-1 upon CHIKV infection (Supplementary Fig. S3). In contrast, when infected monocytes could exchange soluble factors with other leukocytes, a significant increase (p < 0.5) in MCP-1production was observed (Fig. 2d left panel). In effect, the increase in the chemokine levels was comparable to that found in the infected PBMCs of the corresponding donors (Fig. 2d right panel), although the additive role of the cell-to cell contact cannot be excluded. Notably, the increase of MCP-1 was observed regardless of whether MoΔPBMCs were exposed to the virus or not. Altogether, these data suggest that CHIKV infection of monocytes triggers a communication loop with other leukocytes that ultimately leads to MCP-1 upregulation and production in monocytes.

### Monocytes require IFNβ-mediated communication with other leukocytes to produce MCP- 1 in response to CHIKV infection

**Figure 3:**
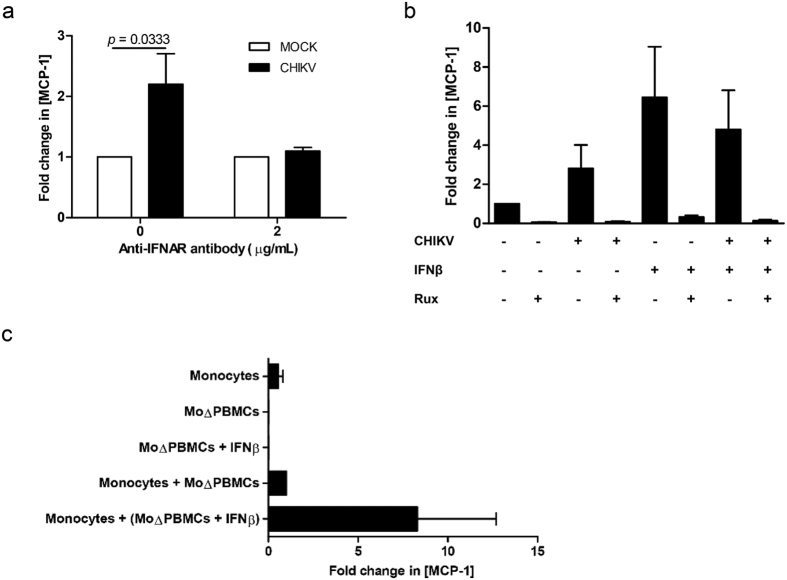
Monocytes require IFNβ-mediated communication with other leukocytes to produce MCP- 1 in response to CHIKV infection. (**a**) Treatment of PBMCs with αIFNAR1/2 antibody abolished CHIKV-mediated MCP-1 production. Bars represent mean + s.e.m., n = 3. (**b**) Treatment of PBMCs with JAK inhibitor Ruxolitinib (Rux) reduced IFNβ- and CHIKV-mediated MCP-1 production. Bars represent mean + s.e.m., n ≥ 2. (**c**) Addition of IFNβ-pretreated MoΔPBMCs to monocytes triggered MCP-1 production measured 24 h post co-culture. Bars represent mean + s.e.m., n = 2.

**Figure 4:**
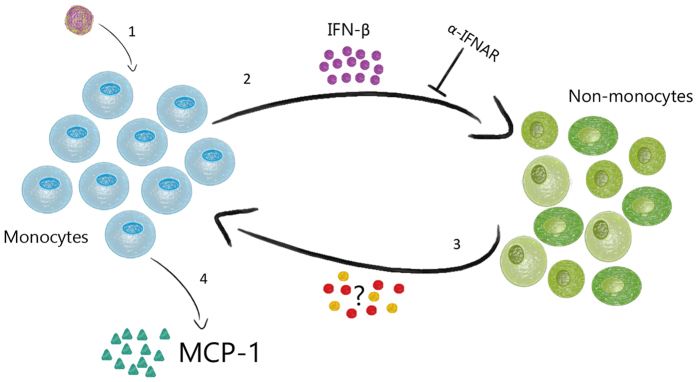
Model of MCP-1 upregulation upon chikungunya virus infection in human PBMCs. (1) CHIKV infection of monocytes triggers (2) the production of IFNβ. (3) Other leukocytes are stimulated by IFNβ and produce (4) one or a combination of soluble factors that stimulates MCP-1 expression in monocytes.

Next, we sought to disclose the soluble factor involved in MCP-1 upregulation. It has been proposed that IFN type I can modulate MCP-1 secretion in a concentration- dependent manner^23^,^40^. Type I IFNs are also readily produced in course of CHIKV infection^37^. Analysis of SN of PBMCs and monocytes revealed that CHIKV infection triggers MOI-dependent production of IFNβ but not that of IFNα (Supplementary Figs S4 and S5). Therefore, we hypothesized that IFNβ stimulation via IFNαβ receptor (IFNAR) played a role in CHIKV infection mediated MCP-1 production in PBMCs. Indeed, pre-incubation of these cells with αIFNAR1/2 antibody abolished CHIKV-mediated MCP-1 production (Fig. 3a). A similar effect was achieved when JAK1/2-STAT signalling downstream of IFNAR was inhibited by Ruxolitinib (Rux) during CHIKV infection and/or IFNβ stimulation (Fig. 3b). Together, these results strongly suggest that IFNβ-mediated signalling is responsible for the MCP-1 production in course of the infection. To substantiate the role of IFNβ in the communication between monocytes and other leukocytes, we next tested whether stimulation of MoΔPBMCs with IFNβ alone for 4 hours prior to their co-culture with monocytes could induce MCP-1 secretion in monocytes. Indeed, as shown in Fig. 3c, addition of IFNβ-pre-treated MoΔPBMCs (washed cells) to monocyte monoculture increased levels of the chemokine after 24 hours of co-culture. As expected, stimulation of MoΔPBMCs with IFNβ did not result in MCP-1 production in the absence of monocytes. In summary, we propose a model of IFNβ-driven communication of infected monocytes with other leukocytes that is essential for MCP-1 secretion upon CHIKV infection in PBMCs (Fig. 4).

### Modulation of MCP-1 does not affect virus replication

**Figure 5:**
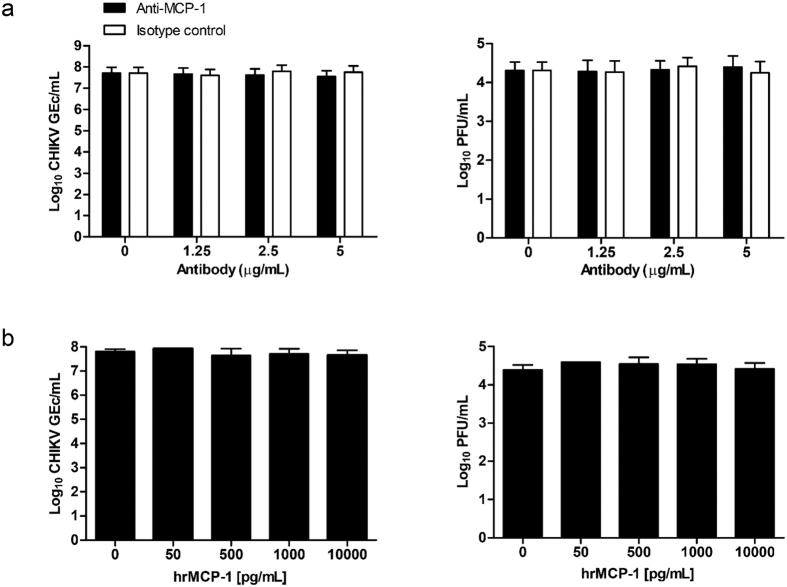
Modulation of MCP-1 levels in the course of infection had no effect on CHIKV production. (**a**) Neutralization of MCP-1 before and during CHIKV infection of PBMCs had no effect on virus replication. Bars represent mean + s.e.m., n = 2. (**b**) CHIKV infection in the presence of increasing concentrations of human recombinant MCP-1 (hrMCP-1) had no effect on virus replication. Bars represent mean + s.e.m., n = 2.

*In vivo*, the increase of levels of MCP-1 coincides with the increase in CHIKV titres^11^,^13^,^36^. Moreover, neutralizing MCP-1 has been shown to induce interferon-stimulated genes (ISGs) including ISG15 which is known to directly affect CHIKV infection^41^,^42^,^43^. Therefore, we investigated whether modulation of this chemokine influences CHIKV replication in PBMCs. Accordingly, we either neutralized endogenous MCP-1 levels by means of an anti-MCP-1 antibody (Supplementary Fig. S6) or performed infection in the presence of increasing concentrations of human recombinant MCP-1 (hrMCP-1) as described in Methods section. Genome-equivalent copies (GEc) and infectious (PFU) titres measured on the supernatants of cells recovered 24 hpi are shown for both treatments. As evidenced by Fig. 4 neither the neutralization (Fig. 5a) nor the addition of MCP-1 (Fig. 5b) had a modulating effect on CHIKV production.

## Discussion

In this study we examined the role and the mechanism of MCP-1 production in CHIKV infection of PBMCs. Our data demonstrates that monocytes are the primary source of MCP-1 during CHIKV infection. Monocytes require IFNβ-mediated communication with other leukocytes for MCP-1 production (Fig. 4). In addition, we show that MCP-1 had no direct effect on the levels of virus progeny.

MCP-1 secretion following active CHIKV infection in PBMCs was dependent on IFNβ. This is in line with the study by Pattison and colleagues, which showed IFNβ is required to sustain MCP-1 production in response to TLR3 activation in bone marrow derived macrophages (BMDMs)^24^. Also, in monocytes IFNβ can stimulate MCP-1 production^23^. Yet, despite the detectable levels of IFNβ following CHIKV infection of monocytes at high MOI (Supplementary Fig. S5), we found no MCP-1 production in these cells, which might indicate that the autocrine effect of IFNβ was blocked during CHIKV infection. This phenomenon could, at least in part, be explained by the fact that CHIKV impedes the ability of infected cells to respond to type I interferon by preventing IFN-induced gene expression^44^. It remains to be seen why non-infected, bystander monocytes did not secrete MCP-1.

Infected monocytes required IFNβ/IFNAR mediated communication with other leukocytes to secrete MCP-1 upon CHIKV infection. In fact, IFNβ-stimulated MoΔPBMCs were able to trigger MCP-1 production in monocytes. Hence, MCP-1 secretion was regulated by yet an unknown soluble factor or factors triggered downstream of IFNAR signalling. MCP-1 levels are known to be controlled by several cytokines including, TNF-α, IL-10, IL-1β and IL-6^21^,^23^,^25^,^45^. Indeed, in our experiments, addition of IL-6 but not CHIKV infection stimulated MCP-1 production in monocytes. This suggests that IL-6 was either not produced by monocytes upon infection, or it was present in amounts insufficient to trigger MCP-1 amplification loop^46^. In fact, lack of IL-6 (and MCP-1) production in CHIKV-infected monocytes, despite its presence in *in vitro* infected whole blood samples, was also reported by Her and colleagues^37^. We therefore deduced that IL-6 is one of the IFNβ-stimulated cytokines responsible for MCP-1 production, and that it is produced by cells other than monocytes. Counterintuitively however, treatment of PBMCs with Ruxolitinib prior to addition of IFNβ, increased the levels of IL-6 (Ruiz Silva, unpublished) while abolishing MCP-1 production completely. Our ongoing studies focus at the disclosure of the identity and the source of IFNβ-stimulated soluble mediator(s) that govern the MCP-1 upregulation in monocytes upon CHIKV infection in PBMCs.

Several viruses induce MCP-1 expression upon infection and it has been shown that the chemokine promotes replication of human immunodeficiency virus (HIV) in macrophages^42^,^47^. Here we showed for the first time that despite the apparent correlation between MCP-1 and CHIKV titre early in infection^4^,^17^,^36^, this chemokine does not play a direct role in CHIKV replication. Yet, it is important to note that in our experimental set up no influx of new target cells was possible. Thus, we cannot rule out a scenario, in which increased levels of MCP-1 in the circulatory system result in an augmented recruitment of monocytes from bone marrow and thereby in an increased pool of cells susceptible to infection^29^,^48^,^49^. Indeed, in mice, inhibition of MCP- 1 with bindarit led to significantly reduced titre of CHIKV at the site of infection^34^. On the other hand, it has been previously suggested that IFNβ-stimulated increase of MCP-1 can contribute to the MCP-1-mediated inhibition of the CCR2 expression and thereby reduce the responsiveness of monocytes to this chemokine^50^. Future research should evaluate whether chemokine and migration receptors are differentially regulated in CHIKV infected vs bystander cells.

In conclusion, our data discloses the complexity of MCP-1 regulation in PBMCs upon CHIKV infection. The crucial role of IFNβ in the induction of MCP-1 in monocytes suggests that balance between these cytokines may be important for an appropriate host response to CHIKV infection.

## Methods

### Cells

Vero E6 (a gift from Dr. G. Pijlman, Wageningen University) and Vero WHO (ATCC) were cultured in DMEM (Life Technologies) containing 10% Fetal Bovine Serum (FBS), penicillin (100 U/ml), streptomycin (100 μg/ml), 10 mM HEPES, and 200 mM glutamine. PBMCs were maintained in RPMI 1640 medium supplemented with 10% FBS. Human PBMCs were isolated from Buffy coats using standard density gradient centrifugation procedures with Ficoll-Paque PLUS (GE Healthcare), as described previously. The buffy coats were obtained from healthy volunteers with informed consent from Sanquin blood bank, in line with the declaration of Helsinki. The PBMCs were cryopreserved at −150 °C.

### Isolation/Depletion of CD14^+^ monocytes

Monocytes were isolated from thawed PBMCs using the Magnisort human CD14 positive selection kit (eBioscience). Briefly, a single-cell suspension containing 1 × 10^8^ PBMCs per mL of cell separation buffer (PBS, 3% FBS, 10mM EDTA) was prepared. Cells were then incubated for 10 min with 20 μL of anti-human CD14 Biotin per 100 μL of cell suspension. Cells were washed and resuspended in separation buffer. Then 30 μL of Magnisort Beads per 100 μL of cell suspension were added. Following 10 min of incubation a magnet was used to remove the bead-bound CD14^+^ cells from the remaining PBMCs. The unbound CD14^-^ cells were also collected. Isolation efficiency was determined by flow cytometry staining with anti-human CD4-PE and anti-human CD14-APC (both, eBioscience).

### Virus and virus titrations

CHIKV (La Reunion OPY1) was a gift from A. Merits (University of Tartu, Estonia), and was produced from infectious cDNA clones and passaged twice in Vero E6 cells^51^. Virus preparations were analysed with respect to the infectious titre and the number of genome equivalents copies, as described previously^52^. Briefly, the infectious virus titre was determined by standard plaque assay on Vero-WHO cells at 37 °C and reverse transcriptase quantitative PCR (RT-qPCR) was used to determine the number of genome equivalents copies (GEc).

Virus inactivation was obtained by 1.5 h incubation of virus aliquots under UVS-28 8 watt Lamp. Inactivation to below level of detection 35 PFU/mL was assessed using standard plaque assay in Vero-WHO cells.

### Intracellular staining

PBMCs (6 × 10^5^ cells/ well) were thawed in RPMI 1640 medium supplemented with 5% FBS and incubated at 37 °C with CHIKV at the indicated MOI. 2 hpi the viral inoculum was removed and the cells were resuspended in complete media and incubated at 37 °C for 18 h. Brefeldin A (Life Technologies) was added 20 hpi to a final concentration of 10 μg/mL and 4 h later (24 hpi) the cells were collected and stained with LIVE/DEAD Fixable Violet Dead Cell Stain (ThermoFisher Scientific), anti-human CD19-FITC and anti-human CD14-APC (both, eBioscience). Following fixation and permeabilization cells were intracellularly stained with anti-human MCP-1-PE (eBioscience). MCP-1 expression was analysed by flow cytometry.

### Inhibition of JAK1/2 signalling

PBMCs were thawed in RPMI 1640 medium supplemented with 5% FBS and pretreated with Ruxolitinib (5 μM, Invivogen) for 2 h at 37 °C. Then the cells were infected with CHIKV at the indicated MOI and/or stimulated with IFNβ (500 U/mL, eBioscience). Ruxolitinib was added to maintain the 5 μM concentration during infection. After 2 h incubation at 37 °C, the inoculum was removed and fresh medium containing IFNβ (500 U/mL) or Ruxolitinib (5 μM) was added to the cells. At 24 hpi, cell-free supernatant was collected and cytokine production was analysed by ELISA.

### Blocking of IFNAR

PBMCs were infected (MOI 1, 5 and 10) in the presence of IFN-α/β receptor chain 2 neutralizing antibody (1, 2 or 5 μg/mL, Mab 1155, clone MMHAR-2; Millipore) for 2 h at 37 °C. The inoculum was removed and fresh medium containing the corresponding antibody concentration was added to the cells. At 24 hpi, cell-free supernatant was collected and cytokine production was analysed by ELISA.

### Co-culture of monocytes with IFNβ pre-stimulated Mo∆PBMCs

Immediately after isolation, 1 × 10^5^ Mo∆PBMCs were incubated for 4 h with IFNβ (500 U/mL). Next, the cells were washed and added to the same amount of unstimulated monocytes. After 24h of co-culture cell-free supernatant was collected and cytokine production was analysed by ELISA as described here below.

### Time course analysis

After 2 h incubation at 37 °C, the inoculum was removed and fresh medium was added to the cells. For all donors tested, cell-free supernatants were collected at each indicated time point, divided into 2 aliquots and stored for subsequent analyses of cytokine and virus production, respectively.

### Co-culture experiments in transwell system

2 hours prior to co-culture, cells were infected at the indicated MOI (1, 5 or 10). After removal of the inoculum cells were co-cultured with 1 × 10^5^ infected, mock-infected or cells treated with 10 ng/μL human recombinant IL-6 (Bio-Connect) in a final volume of 200 μL in 96-transwell plates (0,4 μm pore size, Corning). Monocytes were always placed at the bottom compartment while Mo∆PBMCs were placed at the top compartment of the well. Cell-free supernatants were collected 24 hpi and MCP-1 concentration was determined by ELISA as described below.

### Addition of hrMCP-1

PBMCs were infected in the presence hrMCP-1 (50 to 10000 pg/mL; R&D Systems). 2 hpi the viral inoculum was removed and replaced by fresh medium containing hrMCP-1 to maintain the treatment concentration. At 24 hpi, cell-free supernatant was collected and viral production was determined by plaque assay and qPCR.

### Neutralization

PBMCs were pretreated with different concentrations (1.25, 2.5 and 5 μg/mL) of anti-MCP-1 antibody (clone 5D3-F7; eBioscience) or an isotype control (mouse IgG1 K; eBioscience). The following day (20 hpi) the cells were infected with CHIKV at MOI 1, 5 and 10. 2 hpi the viral inoculum was removed and replaced by fresh medium containing the corresponding antibody. At 24 hpi, cell-free supernatant was collected and viral production was determined by plaque assay and qPCR.

### Flow cytometry

To measure the number of infected cells, PBMCs were fixed at 24 or 48 hpi, and stained using CHIKV E1- specific rabbit antibody (a kind gift from Dr. G. Pijlman) and secondary chicken anti-rabbit AF647 (Life Technologies). Data acquisition was performed on a BD FACSVerse flow cytometer (Becton, Dickinson). Data was analyzed using Kaluza 1.2 (Beckman Coulter).

### Determination of MCP-1 concentration

MCP-1 and other cytokines (including IFNα, IFNβ, IL-6) levels were measured in cell-free supernatants using hMCP-1 Ready-steady-Go ELISA and ProcartaPlex (both from eBioscences) according to respective manufacturer’s instructions.

### Statistics

All data are expressed as mean with bars representing standard error of the mean (s.e.m) (unless specified). Unless indicated one-tailed unpaired student’s *t-*test was used for analysis in GraphPad Prism 5 application. Values of *p < 0.05, **p < 0.01 and ***p < 0.001 were considered significant.

## Additional Information

**How to cite this article**: Ruiz Silva, M. *et al.* Mechanism and role of MCP-1 upregulation upon chikungunya virus infection in human peripheral blood mononuclear cells. *Sci. Rep.* **6**, 32288; doi: 10.1038/srep32288 (2016).

## Supplementary Material

Supplementary Information

## References

[b1] Pan American Health Organization, WHO. Number of reported cases of Chikungunya Fever in the Americas. (2014–2016) (Date of access: 13/05/2016) http://www.paho.org/hq/index.php?option=com_topics&view=readall&cid=5927&Itemid=40931&lang=en.

[b2] Dupuis-Maguiraga L. *et al.* Chikungunya disease: infection-associated markers from the acute to the chronic phase of arbovirus-induced arthralgia. PLoS Negl Trop Dis 6, e1446 (2012). 22479654 10.1371/journal.pntd.0001446 PMC3313943

[b3] Suhrbier A., Jaffar-Bandjee M. & Gasque P. Arthritogenic alphaviruses–an overview. Nature Reviews Rheumatology 8, 420–429 (2012). 22565316 10.1038/nrrheum.2012.64

[b4] Hoarau J. J. *et al.* Persistent chronic inflammation and infection by Chikungunya arthritogenic alphavirus in spite of a robust host immune response. J. Immunol. 184, 5914–5927 (2010). 20404278 10.4049/jimmunol.0900255

[b5] Cardona-Ospina J. A., Henao-SanMartin V., Paniz-Mondolfi A. E. & Rodríguez-Morales A. J. Mortality and fatality due to Chikungunya virus infection in Colombia. Journal of Clinical Virology 70, 14–15 (2015). 26305812 10.1016/j.jcv.2015.07.001

[b6] Rollé A. *et al.* Severe Sepsis and Septic Shock Associated with Chikungunya Virus Infection, Guadeloupe, 2014. Organ 15, 0.001 (2016). 10.3201/eid2205.151449 PMC4861514 27088710

[b7] Gerardin P. *et al.* Chikungunya virus-associated encephalitis: A cohort study on La Reunion Island, 2005–2009. Neurology 86, 94–102 (2016). 26609145 10.1212/WNL.0000000000002234

[b8] Rajapakse S., Rodrigo C. & Rajapakse A. Atypical manifestations of chikungunya infection. Trans. R. Soc. Trop. Med. Hyg. 104, 89–96 (2010). 19716149 10.1016/j.trstmh.2009.07.031

[b9] Sharp T. *et al.* 1975Clinicopathologic Characteristics and Immunolocalization of Viral Antigens in Chikungunya-Associated Fatal Cases–Puerto Rico, 2014 (Open Forum Infectious Diseases Ser. 2, Oxford University Press, 2015).

[b10] Couderc T. *et al.* A mouse model for Chikungunya: young age and inefficient type-I interferon signaling are risk factors for severe disease. PLoS Pathog 4, e29 (2008). 18282093 10.1371/journal.ppat.0040029 PMC2242832

[b11] Chow A. *et al.* Persistent arthralgia induced by Chikungunya virus infection is associated with interleukin-6 and granulocyte macrophage colony-stimulating factor. J. Infect. Dis. 203, 149–157 (2011). 21288813 10.1093/infdis/jiq042 PMC3071069

[b12] Kelvin A. A. *et al.* Inflammatory cytokine expression is associated with chikungunya virus resolution and symptom severity. PLoS Negl Trop Dis 5, e1279 (2011). 21858242 10.1371/journal.pntd.0001279 PMC3156690

[b13] Reddy V., Mani R. S., Desai A. & Ravi V. Correlation of plasma viral loads and presence of Chikungunya IgM antibodies with cytokine/chemokine levels during acute Chikungunya virus infection. J. Med. Virol. 86, 1393–1401 (2014). 24523146 10.1002/jmv.23875

[b14] Venugopalan A., Ghorpade R. P. & Chopra A. Cytokines in acute chikungunya. PloS one 9, e111305 (2014). 25343623 10.1371/journal.pone.0111305 PMC4208842

[b15] Poo Y. S. *et al.* Multiple immune factors are involved in controlling acute and chronic chikungunya virus infection. PLoS Negl Trop Dis 8, e3354 (2014). 25474568 10.1371/journal.pntd.0003354 PMC4256279

[b16] Labadie K. *et al.* Chikungunya disease in nonhuman primates involves long-term viral persistence in macrophages. J. Clin. Invest. 120, 894–906 (2010). 20179353 10.1172/JCI40104 PMC2827953

[b17] Gardner J. *et al.* Chikungunya virus arthritis in adult wild-type mice. J. Virol. 84, 8021–8032 (2010). 20519386 10.1128/JVI.02603-09 PMC2916516

[b18] Chen W. *et al.* Arthritogenic alphaviral infection perturbs osteoblast function and triggers pathologic bone loss. Proc. Natl. Acad. Sci. USA 111, 6040–6045 (2014). 24733914 10.1073/pnas.1318859111 PMC4000821

[b19] Dhanwani R. *et al.* Characterization of Chikungunya virus induced host response in a mouse model of viral myositis. PloS one 9, e92813 (2014). 24667237 10.1371/journal.pone.0092813 PMC3965460

[b20] Deshmane S. L., Kremlev S., Amini S. & Sawaya B. E. Monocyte chemoattractant protein-1 (MCP-1): an overview. J. Interferon Cytokine Res. 29, 313–326 (2009). 19441883 10.1089/jir.2008.0027 PMC2755091

[b21] Biswas P. *et al.* Interleukin-6 induces monocyte chemotactic protein-1 in peripheral blood mononuclear cells and in the U937 cell line. Blood 91, 258–265 (1998). 9414293

[b22] Ciesielski C. J., Andreakos E., Foxwell B. M. & Feldmann M. TNFα‐induced macrophage chemokine secretion is more dependent on NF‐κB expression than lipopolysaccharides‐induced macrophage chemokine secretion. Eur. J. Immunol. 32, 2037–2045 (2002). 12115625 10.1002/1521-4141(200207)32:7<2037::AID-IMMU2037>3.0.CO;2-I

[b23] Fantuzzi L., Canini I., Belardelli F. & Gessani S. IFN-beta stimulates the production of beta-chemokines in human peripheral blood monocytes. Importance of macrophage differentiation. Eur. Cytokine Netw. 12, 597–603 (2001). 11781186

[b24] Pattison M. J., MacKenzie K. F., Elcombe S. E. & Arthur J. S. C. IFNβ autocrine feedback is required to sustain TLR induced production of MCP-1 in macrophages. FEBS Lett. 587, 1496–1503 (2013). 23542035 10.1016/j.febslet.2013.03.025 PMC3655261

[b25] Steube K. G., Meyer C. & Drexler H. G. Constitutive protein expression of monocyte chemotactic protein-1 (MCP-1) by myelomonocytic cell lines and regulation of the secretion by anti- and proinflammatory stimuli. Leuk. Res. 23, 843–849 (1999). 10475624 10.1016/s0145-2126(99)00107-1

[b26] Shi C. & Pamer E. G. Monocyte recruitment during infection and inflammation. Nature Reviews Immunology 11, 762–774 (2011). 10.1038/nri3070 PMC3947780 21984070

[b27] Held K. S., Chen B. P., Kuziel W. A., Rollins B. J. & Lane T. E. Differential roles of CCL2 and CCR2 in host defense to coronavirus infection. Virology 329, 251–260 (2004). 15518805 10.1016/j.virol.2004.09.006 PMC7111831

[b28] Dessing M. C., van der Sluijs, Koenraad F., Florquin S. & van der Poll T. Monocyte chemoattractant protein 1 contributes to an adequate immune response in influenza pneumonia. Clinical immunology 125, 328–336 (2007). 17827068 10.1016/j.clim.2007.08.001

[b29] Bardina S. V. *et al.* Differential Roles of Chemokines CCL2 and CCL7 in Monocytosis and Leukocyte Migration during West Nile Virus Infection. J. Immunol. 195, 4306–4318 (2015). 26401006 10.4049/jimmunol.1500352 PMC4610864

[b30] Lu B. *et al.* Abnormalities in monocyte recruitment and cytokine expression in monocyte chemoattractant protein 1-deficient mice. J. Exp. Med. 187, 601–608 (1998). 9463410 10.1084/jem.187.4.601 PMC2212142

[b31] Poo Y. S. *et al.* CCR2 deficiency promotes exacerbated chronic erosive neutrophil-dominated chikungunya virus arthritis. J. Virol. 88, 6862–6872 (2014). 24696480 10.1128/JVI.03364-13 PMC4054367

[b32] Lim J. K. *et al.* Chemokine receptor Ccr2 is critical for monocyte accumulation and survival in West Nile virus encephalitis. J. Immunol. 186, 471–478 (2011). 21131425 10.4049/jimmunol.1003003 PMC3402345

[b33] Dawson T. C., Beck M. A., Kuziel W. A., Henderson F. & Maeda N. Contrasting effects of CCR5 and CCR2 deficiency in the pulmonary inflammatory response to influenza A virus. The American journal of pathology 156, 1951–1959 (2000). 10854218 10.1016/S0002-9440(10)65068-7 PMC1850091

[b34] Chen W. *et al.* Bindarit, an inhibitor of monocyte chemotactic protein synthesis, protects against bone loss induced by chikungunya virus infection. J. Virol. 89, 581–593 (2015). 25339772 10.1128/JVI.02034-14 PMC4301140

[b35] Rulli N. E. *et al.* Protection from arthritis and myositis in a mouse model of acute chikungunya virus disease by bindarit, an inhibitor of monocyte chemotactic protein-1 synthesis. J. Infect. Dis. 204, 1026–1030 (2011). 21881117 10.1093/infdis/jir470

[b36] Teng T. S. *et al.* A Systematic Meta-analysis of Immune Signatures in Patients With Acute Chikungunya Virus Infection. J. Infect. Dis. 211, 1925–1935 (2015). 25635123 10.1093/infdis/jiv049 PMC4442625

[b37] Her Z. *et al.* Active infection of human blood monocytes by Chikungunya virus triggers an innate immune response. J. Immunol. 184, 5903–5913 (2010). 20404274 10.4049/jimmunol.0904181

[b38] Yoshimura T. *et al.* Human monocyte chemoattractant protein‐1 (MCP‐1) Full‐length cDNA cloning, expression in mitogen‐stimulated blood mononuclear leukocytes, and sequence similarity to mouse competence gene JE. FEBS Lett. 244, 487–493 (1989). 2465924 10.1016/0014-5793(89)80590-3

[b39] Zaritsky L., Bedsaul J. & Zoon K. I. D. 30: Virus MOI affects type I IFN subtype induction profiles and ISGs. Cytokine 76, 69 (2015). 10.1128/JVI.01727-15 PMC4645643 26355085

[b40] Comabella M., Imitola J., Weiner H. L. & Khoury S. J. Interferon-β treatment alters peripheral blood monocytes chemokine production in MS patients. J. Neuroimmunol. 126, 205–212 (2002). 12020972 10.1016/s0165-5728(02)00064-4

[b41] Werneke S. W. *et al.* ISG15 is critical in the control of Chikungunya virus infection independent of UbE1L mediated conjugation. PLoS Pathog 7, e1002322 (2011). 22028657 10.1371/journal.ppat.1002322 PMC3197620

[b42] Sabbatucci M. *et al.* Endogenous CCL2 neutralization restricts HIV-1 replication in primary human macrophages by inhibiting viral DNA accumulation. Retrovirology 12, 4-014-0132-6 (2015). 10.1186/s12977-014-0132-6 PMC4314729 25608886

[b43] Teng T. S. *et al.* Viperin restricts chikungunya virus replication and pathology. J. Clin. Invest. 122, 4447–4460 (2012). 23160199 10.1172/JCI63120 PMC3533538

[b44] Fros J. J. *et al.* Chikungunya virus nonstructural protein 2 inhibits type I/II interferon-stimulated JAK-STAT signaling. J. Virol. 84, 10877–10887 (2010). 20686047 10.1128/JVI.00949-10 PMC2950581

[b45] Harigai M. *et al.* Monocyte chemoattractant protein-1 (MCP-1) in inflammatory joint diseases and its involvement in the cytokine network of rheumatoid synovium. Clin. Immunol. Immunopathol. 69, 83–91 (1993). 8403545 10.1006/clin.1993.1153

[b46] Roca H. *et al.* CCL2 and interleukin-6 promote survival of human CD11b+ peripheral blood mononuclear cells and induce M2-type macrophage polarization. J. Biol. Chem. 284, 34342–34354 (2009). 19833726 10.1074/jbc.M109.042671 PMC2797202

[b47] Ansari A. W., Heiken H., Meyer-Olson D. & Schmidt R. E. CCL2: a potential prognostic marker and target of anti-inflammatory strategy in HIV/AIDS pathogenesis. Eur. J. Immunol. 41, 3412–3418 (2011). 22076814 10.1002/eji.201141676

[b48] Smith M. S., Bentz G. L., Alexander J. S. & Yurochko A. D. Human cytomegalovirus induces monocyte differentiation and migration as a strategy for dissemination and persistence. J. Virol. 78, 4444–4453 (2004). 15078925 10.1128/JVI.78.9.4444-4453.2004 PMC387677

[b49] Fantuzzi L., Belardelli F. & Gessani S. Monocyte/macrophage-derived CC chemokines and their modulation by HIV-1 and cytokines: a complex network of interactions influencing viral replication and AIDS pathogenesis. J. Leukoc. Biol. 74, 719–725 (2003). 12960239 10.1189/jlb.0403175

[b50] Fantuzzi L. *et al.* Loss of CCR2 expression and functional response to monocyte chemotactic protein (MCP-1) during the differentiation of human monocytes: role of secreted MCP-1 in the regulation of the chemotactic response. Blood 94, 875–883 (1999). 10419877

[b51] Pohjala L. *et al.* Inhibitors of alphavirus entry and replication identified with a stable Chikungunya replicon cell line and virus-based assays. PLoS One 6, e28923 (2011). 22205980 10.1371/journal.pone.0028923 PMC3242765

[b52] van Duijl-Richter M. K., Blijleven J. S., van Oijen A. M. & Smit J. M. Chikungunya virus fusion properties elucidated by single-particle and bulk approaches. J. Gen. Virol. 96, 2122–2132 (2015). 25872739 10.1099/vir.0.000144

