# Supplementary material for: Mechanism and role of MCP-1 upregulation upon chikungunya virus infection in human peripheral blood mononuclear cells
Source: Sci Rep. 2016 Aug 25;6:32288. doi: 10.1038/srep32288 (PMC4997611; doi:10.1038/srep32288)

Supplementary Information

Mechanism and role of MCP-1 upregulation upon chikungunya virus infection in human peripheral blood mononuclear cells

Mariana Ruiz Silva, Heidi van der Ende-Metselaar, H. Lie Mulder, Jolanda M. Smit, Izabela A. Rodenhuis-Zybert*

Department of Medical Microbiology, University of Groningen and University Medical Center Groningen, 9700 RB Groningen, The Netherlands

***Corresponding author**: i.a.rodenhuis-zybert@umcg.nl

Supplementary Figure S1. Viability and CD14 expression of PBMCs upon CHIKV infection**.** Left. Viability of PBMCs measured 24h after CHIKV infection. Right. CD14 expression relative to mock-infected cells. Data represent mean fold change + s.e.m, n=2.

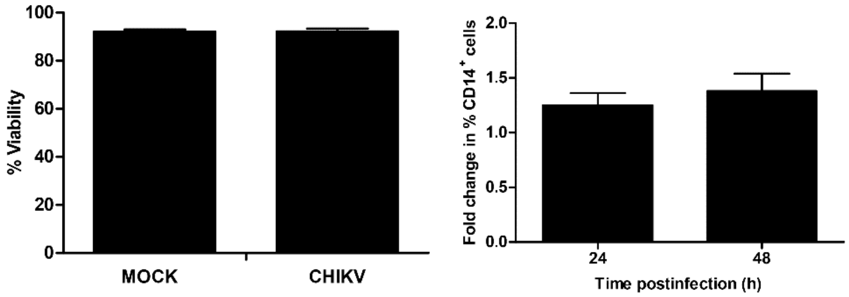

Supplementary Figure S2. MCP-1 levels determined in the supernatant from CHIKV-infected monocytes 24 hpi. Data represent mean fold change with respect to mock-infection + s.e.m, n≥2.

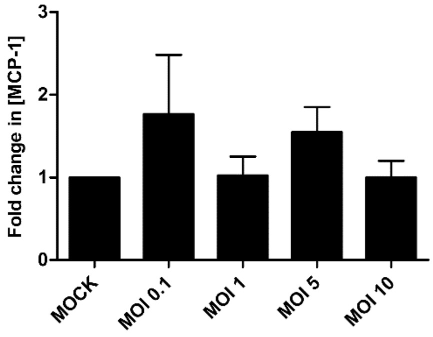

Supplementary Figure S3. MCP-1 levels determined in supernatant from CHIKV-infected cells 24 hpi. Data represent mean fold change with respect to mock-infection of (Monocytes + MoΔPBMCs) + s.e.m, n=3.

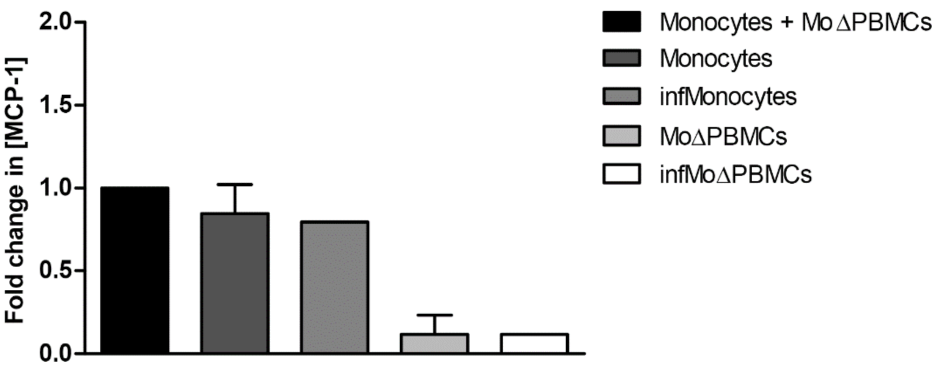

Supplementary Figure S4. IFNα and IFNβ release by PBMCs at 24 h after infection with CHIKV. Data represent the mean fold change of secreted type I IFNs with respect to mock-infection ±s.e.m, n=3

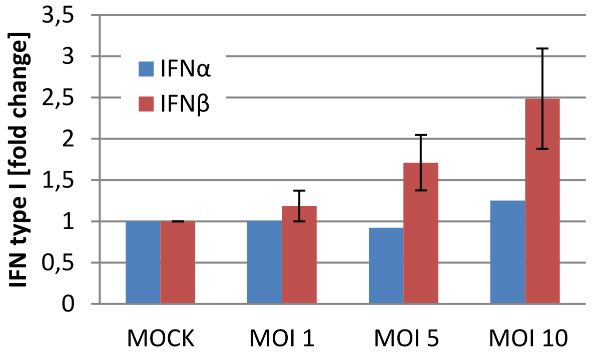

Supplementary Figure S5. IFNα and IFNβ secreted by monocytes at 24 h after infection with CHIKV. Data from one experiment.

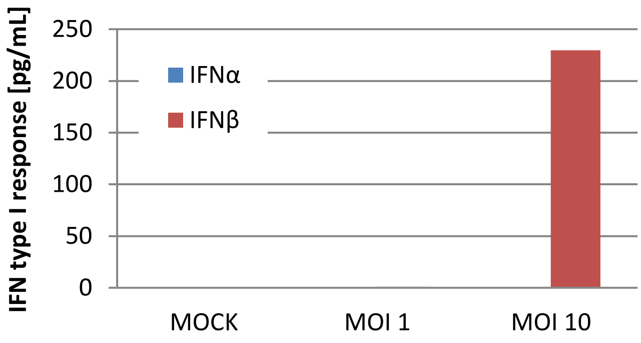

Supplementary Figure S6. Levels of MCP-1 in supernatants of PBMCs treated with anti-MCP-1 neutralizing antibody and infected with CHIKV (MOI 1). Data from one representative experiment.

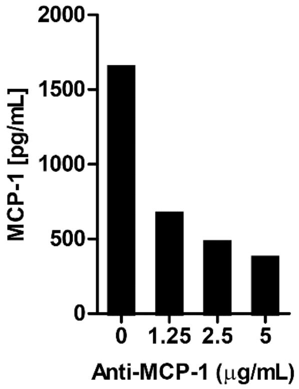

Supplement: Supplementary Information [file srep32288-s1.pdf]
